# Correlation of (1→3)-β-D-glucan with other inflammation markers in chronically HIV infected persons on suppressive antiretroviral therapy

**DOI:** 10.3205/id000018

**Published:** 2015-12-22

**Authors:** Martin Hoenigl, Michelli Faria de Oliveira, Josué Pérez-Santiago, Yonglong Zhang, Steven Paul Woods, Malcolm Finkelman, Sara Gianella

**Affiliations:** 1 Division of Infectious Diseases, University of California San Diego, San Diego, California, USA; 2 Section of Infectious Diseases and Tropical Medicine, Department of Internal Medicine, Medical University of Graz, Graz, Austria; 3 Division of Pulmonology, Department of Internal Medicine, Medical University of Graz, Graz, Austria; 4 Research Laboratory, Associates of Cape Cod, Inc., Falmouth, Massachusetts, USA; 5 Department of Psychology, University of Houston, Texas, USA

**Keywords:** beta-D-glucan, HIV, virologically suppressed, neopterin, plasma, interleukin

## Abstract

We evaluated associations between levels of BDG and other biomarkers of inflammation in blood from 41 virologically suppressed persons with chronic HIV-infection. We found a significant correlation between BDG and neopterin levels (r=0.68), and trends to significance for correlations with other inflammation markers (tumor-necrosis-factor-α: r=0.30; interleukin-8: r=0.30; interleukin-6: r=0.28). In conclusion, BDG levels correlated with inflammation markers in a cohort of virologically suppressed individuals with chronic HIV infection. Future studies are needed to evaluate whether BDG may be a marker for morbidity in chronic HIV infection.

## Introduction

Antiretroviral therapy (ART) can suppress HIV replication for long-term in most HIV-infected individuals who have good adherence to therapy [1]. Although the advent of ART has increased the life expectancy and decreased morbidity of HIV-infected individuals, they still present higher rates of non-AIDS defining disorders, such as cardiovascular disease and neurocognitive impairment, than HIV-uninfected individuals [2]. These events have been associated with residual immune dysfunction which persists in some individuals despite long term suppressive ART [3]. The exact mechanism of chronic immune dysfunction is incompletely understood and most likely multifactorial in origin.

Previous reports have indicated that microbial translocation of bacterial and fungal commensals of the gastrointestinal tract into systemic circulation is one important factor, which is associated with immune dysfunction, persistent inflammation and likely also plays a role in the disease progression, and non-AIDS associated comorbidities [4], [5]. The enteropathy associated with HIV infection is characterized by microbial over-growth in the intestinal lumen and disrupted intestinal permeability resulting in increased levels of lipopolysaccharides (LPS) and 16S rRNA in blood plasma. Such microbial translocation most likely leads to local and systemic immune activation, characterized by increased levels of pro-inflammatory cytokines such as tumour-necrosis-factor α (TNF-α), neopterin, cluster of differentiation 14 (CD14), interleukin-8 (IL-8), and interleukin-6 (IL-6) [6], [7], [8], [9]. In particular, plasma neopterin is an established marker of monocyte activation and was repeatedly associated with HIV disease progression, greater peripheral monocyte HIV DNA reservoirs and negative neurocognitive and cardiovascular outcomes [10], [11], [12].

(1→3)-β-D-glucan (BDG) is a polysaccharide component of the cell wall of *Pneumocystis jirovecii* (PJ), *Candida* spp. and several other fungal species excluding Mucorales and *Cryptococcus* spp. A number of previous studies have shown that blood BDG levels, determined by the Fungitell^®^ assay (Associates of Cape Cod, USA) in serum are useful for early diagnosis of invasive fungal infections (IFI) or PJ-pneumonia (PJP) [13], [14], [15], [16], [17]. In the absence of an active IFI or PJP, serum BDG may be a reasonable indicator of gut mucosal barrier impairment [18], [19] and microbial translocation [20]. The latter was recently reported also for a cohort of HIV-infected subjects [5].

## Methods

In this study we measured plasma BDG and compared levels with those of established biomarkers of immune activation and microbial translocation in a cohort of virologically suppressed individuals with chronic HIV infection. Study samples were collected as part of a prospective study between May 2008 and February 2013 at the University of California, San Diego. Plasma samples were stored at −80°C at the day of collection and 41 samples from 41 subjects with suppressed levels of HIV RNA were randomly selected for retrospective evaluation of BDG levels and other biomarkers. sCD14 (Trillium Diagnostics, Brewer, ME, USA) and neopterin (Thermo Scientific, Waltham, MA, USA) were measured by enzyme-linked immunosorbent assays (ELISAs), while IL-8, IL-6 and TNF-α were measured by electrochemiluminescence multiplex assay (Meso Scale Diagnostics, Rockville, MD, USA), all according to the manufacturer’s procedures. BDG testing of plasma samples was performed in March 2015 at Associates of Cape Cod, Inc., research laboratories using the Fungitell assay (Cape Cod, Inc., East Falmouth, USA).

For statistical analysis SPSS 21 (SPSS Inc., Chicago, IL, USA) was used. BDG levels were squareroot transformed to achieve a distribution close to normal. Correlation between levels of BDG and levels of other biomarkers was calculated using Pearson correlation analysis. The UCSD Human Research Protections Program approved the study protocol, consent and all study related procedures. All study participants provided voluntary, written informed consent before any study procedures were undertaken.

## Results

Median age of the study population was 51 years (range 22–71), 32 participants were males, 9 females. Twenty-six were Caucasian, 9 African-American and 6 reported other race. Median estimated duration of infection was 14.4 years (range 0.4–26.3 years), median CD4 cell count was 643 (range 196–1,740). All participants were virologically suppressed at the time of sampling, with a minority (25%) still being on their first ART regimen. None of the participants had an active fungal infection and none was treated with systemic antifungal agents during the 6 months before participating in the study.

Median BDG level was 15 pg/mL (range: 5–238 pg/mL). BDG levels, levels of other biomarkers and correlations are displayed in Table 1. Higher levels of BDG were associated with higher levels of neopterin (r=0.68; p<0.001). We also found some non-significant trends for positive correlations between BDG and other inflammation markers, while no correlation was found between BDG and sCD14. Results are shown in Table 1. In addition, higher levels of BDG were correlated with higher percentage of neutrophils among white blood cell count (r=0.35, p=0.024). No correlations were found between BDG and age, sex, and estimated duration of infection. BDG was significantly higher in those with a CD4 count below 300 cells/mL (n=4), when compared to those above that threshold (n=37; p<0.001, two-tailed t-test).

## Discussion

We found that BDG levels were low in a cohort of virologically suppressed individuals with chronic HIV infection, but nevertheless correlated strongly with plasma neopterin levels, and also slightly with TNF-α, IL-6, and IL-8 levels. Our results confirm findings of a previous study, that evaluated BDG levels in a cohort of in HIV-infected outpatients (majority not virologically suppressed) and found that plasma IL-8 (p=0.03), and TNF-α (p=0.03) were increased in those with high BDG levels (i.e. >40 pg/mL), while IL-6 was not significantly different [5]. Another study that evaluated BDG as a marker for cryptococcal meningitis among HIV-infected individuals in cerebrospinal fluid (CSF) (21) found positive correlations between BDG and IL-8 (p<0.01), and also TNF-α (p=0.02), while again no correlation was found with IL-6 [21]. The observed correlation of BDG with IL-8 may be explained by findings of another study reporting that (1→3)-β-D-glucans powerfully co-stimulate cytokine production (IL-6/IL-8) [22]. However it is unclear why a similar association was not observed with IL-6.

Our results further indicate that BDG levels are higher in asymptomatic individuals with CD4 counts below 300 cells/mL. Explanations include potential colonization or subclinical infection with *Candida* spp. or *Pneumocystis* that may occur more frequently in individuals with lower CD4 counts [13]. It has been shown previously that BDG levels were markedly higher (mean 142 pg/mL) in a HIV infected cohort with lower median CD4 counts (26, IQR 10–53, all without opportunistic infections), when compared to the cohort studied here (with a median CD4 count >600 pg/mL) [13]. In another study, high serum BDG levels (>40 pg/mL) were more likely to occur in individuals with CD4 counts less than 200 cells/mL (31.8% vs. 8.4%, p<0.01), higher HIV viral levels (2.85 vs. 2.13 log_10_ copies/mL, p<0.01), and those without ART (68.2% vs. 90.0%, p<0.01) [5].

Major limitations of our pilot study include the small sample size. To further examine the role of BDG as a potential biomarker for microbial translocation and its correlation with immune dysfunction and non-AIDS clinical events during HIV infection more comprehensive studies will be necessary. Also BDG levels were determined in plasma samples. While the test is officially licensed for testing of serum samples, previous studies have indicated that detection of BDG in plasma may be associated with similar performance characteristics [14].

## Conclusion

In conclusion, BDG levels correlated with inflammation markers in a cohort of virologically suppressed individuals with chronic HIV infection. Future studies are needed to evaluate whether BDG may be a marker for morbidity in chronic HIV infection.

## Figures and Tables

**Table 1:** Results for all investigated biomarkers [median and (IQR) or mean ± standard deviation (SD) are displayed] and correlation of β-D-glucan (BDG; squareroot transformed to achieve distribution close to normal) with other biomarkers

Biomarkers	Results [median (IQR) or mean ± SD]	Correlation between squareroot BDG and other biomarkers	p-value

BDG (pg/mL)	15 (8–21)	–	–
Square root BDG	4.96 ± 2.33	–	–
TNF-α (pg/mL)	1.82 ± 0.88	r=0.29	0.06
Neopterin (ng/mL)	2.94 ± 1.86	r=0.68	<0.001
IL-8 (pg/mL)	6.31 ± 3.30	r=0.30	0.06
IL-6 (pg/mL)	0.6319 ± 0.4880	r=0.28	0.07
sCD14 (ng/mL)	1,425 ± 650	r=0.19	0.23
